# The Enculturated Move From Proto-Arithmetic to Arithmetic

**DOI:** 10.3389/fpsyg.2019.01454

**Published:** 2019-07-09

**Authors:** Markus Pantsar

**Affiliations:** Department of Philosophy, History and Art University of Helsinki, Helsinki, Finland

**Keywords:** enculturated cognition, arithmetical cognition, proto-arithmetic, philosophy of mathematics, cumulative cultural evolution

## Abstract

The basic human ability to treat quantitative information can be divided into two parts. With proto-arithmetical ability, based on the core cognitive abilities for subitizing and estimation, numerosities can be treated in a limited and/or approximate manner. With arithmetical ability, numerosities are processed (counted, operated on) systematically in a discrete, linear, and unbounded manner. In this paper, I study the theory of enculturation as presented by Menary (2015) as a possible explanation of how we make the move from the proto-arithmetical ability to arithmetic proper. I argue that enculturation based on neural reuse provides a theoretically sound and fruitful framework for explaining this development. However, I show that a comprehensive explanation must be based on valid theoretical distinctions and involve several stages in the development of arithmetical knowledge. I provide an account that meets these challenges and thus leads to a better understanding of the subject of enculturation.

## Introduction

In this paper, I focus on a particular stage in the development of mathematical cognition that is of high general scientific and philosophical importance. There is a growing amount of data suggesting that our first ability to treat observations in terms of quantities comes from core cognitive systems we already possess as infants and share with many nonhuman animals (see, e.g., Dehaene, 1997/2011 and Kadosh and Dowker, 2015 for overviews). According to a widely accepted paradigm, these core cognitive abilities are later developed into systematic ways of processing discrete quantities (Dehaene, 1997/2011; Butterworth, 1999; Spelke, 2000; Halberda and Feigenson, 2008; Carey, 2009). Unlike the emergence of the core cognitive abilities, this step does not occur universally, but most cultures develop some system of numerals to deal at least with small quantities (Ifrah, 1998; Everett, 2017). I call the ability up to and including this point *proto-arithmetic*. Some cultures also develop the treatment of quantities beyond the proto-arithmetical ability. They create recursive number systems which can be used without expressive limits, as well as operations (addition, multiplication, etc.) in these systems. Such number systems I call *arithmetic* proper. The main question here concerns the development from the core cognitive abilities to acquiring proper arithmetical knowledge and skills.

I approach this question both from the ontogenetic and the phylogenetic angle on the basis of Menary’s (2015) theory of enculturation, which refers to the way cognitive processes develop, determined by surrounding cultural input. I will show that the move from proto-arithmetic to arithmetic poses a key question in explaining mathematical cognition, and the enculturation account provides a fruitful framework for analyzing it. In section “What Is Arithmetic?” I start by establishing the difference between proto-arithmetical and arithmetical ability. In section “From Proto-Arithmetic to Arithmetic” I clarify what is involved in the move from proto-arithmetic to arithmetic. This is then analyzed in section “Enculturation as the Answer?” through the enculturation account on the level of physiological and cultural manifestations. In section “Whither Enculturation?” I conclude that while enculturation provides a theoretically and empirically sound and fruitful platform for studying the development of arithmetical knowledge, we are currently only at the very beginning of that journey. I also outline how to relate the enculturation framework to existing and future empirical research in fields like mathematics education.

### What Is Arithmetic?

When we want to clarify the nature and the origin of arithmetical knowledge, the first order of business is to determine what we mean by “arithmetic.” In the mathematical, philosophical, and empirical literature, there are several meanings of the word, which can be problematic when we want to construct mathematically valid and empirically informed philosophical accounts of mathematics. Unfortunately, in the literature on what is usually called “numerical cognition” or “number cognition,” there is a tendency to conflate the meaning of several key concepts. “Number,” “natural number,” “numerosity,” and “quantity” are often used interchangeably in different contexts. In the same discussion, “number” may be used to refer to a term of formal arithmetic, as well as to an infant or animal ability for treating quantities. Thus, when scientists write about “arithmetic,” it is sometimes impossible to determine what is meant. The research direction established by Wynn (1992), for example, is often referred to as “infant arithmetic.” Similarly, empirical scientists have written about “arithmetic in newborn chicks” (Rugani et al., 2009) and “numerical and arithmetical abilities in non-primate species” (Agrillo, 2014).

Were this merely a case of different standards of terminology, the problem would be less serious. However, describing infant and nonhuman animal abilities as “arithmetic” can cause crucial misinterpretations of data. For example, Wynn’s massively influential 1992 paper was called “Addition and subtraction by human infants.” In the abstract of the paper, Wynn writes:

Here I show that 5-month-old infants can calculate the results of simple arithmetical operations on small numbers of items. This indicates that infants possess true numerical concepts, and suggests that humans are innately endowed with arithmetical abilities (Wynn, 1992).

Did the data show that? By reacting to the unnatural situation in which one and one doll equals one doll (the other doll having been removed clandestinely), Wynn argues that the infants showed arithmetical ability by calculating the results of arithmetical operations. However, we can equally well explain the infant behavior by assuming that they only kept track of one quantity, perhaps by a totally different type of cognitive or perceptual mechanism^1^. In a similar manner, there does not seem to be any reason to assume that the infants possess “true numerical concepts.” What the experiment – later replicated many times – did show was that infants have the ability to individuate objects and that they react to unnatural changes in the numerosity of objects. This can be explained without ascribing numerical concepts to the infants. It is therefore important to establish the difference between the fact that we can characterize behavior in arithmetical terms and that behavior being truly based on arithmetical ability. Referring to the infants’ surprise to the “addition” 1 + 1 = 1 may be an illuminating way to describe their behavior. But it should not be confused with thinking that infants did the addition 1 + 1 = 2 and were surprised when the result did not match their observations. Thus, the terminological issue becomes an important one for the postulates made in a theory. In the worst case, the use of arithmetical terminology may determine what kind of abilities we assume subjects (such as infants and nonhuman animals) to possess.

For this reason, before we start explaining what kind of cognitive and cultural aspects are involved in the development of arithmetical knowledge, we must make clear what we are explaining. This is not a straight-forward matter, since even in the developed mathematical sense there are different meanings for the term “arithmetic” in the literature. In the philosophy of mathematics, arithmetic is often understood in terms of formal systems like first-order Peano arithmetic. From an empirically informed perspective, however, that appears to be a needlessly limited interpretation. When we want to explain the development of arithmetical knowledge, the introduction of formal axiomatic systems is a very late development that was preceded by a long history of successful use of arithmetical operations as well as explicit arithmetical proofs. We certainly do not want to confuse arithmetic with the primitive ability with quantities that infants and nonhuman animals have, but it is equally important not to give too strict criteria for arithmetic and end up accepting only modern formal systems as suitable referents of the term in philosophical contexts.

Therefore, I suggest here a definition of arithmetic as a *sufficiently rich discrete linear system of explicit number words or symbols with specified rules of operations*. What counts as sufficiently rich cannot be defined uniformly and it depends on the numeral system. The key idea here is that for a system to count as arithmetical, it has to follow the standard ordering of the set of natural numbers (the omega progression) sufficiently closely to allow grasping the essential structure of that progression. If, for example, a child is able to add and multiply numbers larger than 100, it seems likely that she has grasped something essential about the natural numbers. Such ability cannot be achieved through rote memorization of multiplication tables, which might involve little understanding of the structure of the natural number system. Put more precisely, in arithmetical systems, numerals (or number symbols) need to follow a distinct *recursive* structure, which is grasped by competent users of the systems. In the simplest case, a recursive numeral system can contain only one symbol. If N denoted the numerosity “one,” simply by adding Ns, we can get a recursive numeral system. NN denotes “two,” NNN “three,” and so on. Such numeral systems are cumbersome and difficult to communicate verbally, so all numeral systems in natural languages have more words. Thus, they start showing recursivity later on in the numeral sequence. Numerals in the English language, for example, start showing recursive structure after 12. Up to that point, the numeral names must simply be memorized individually. In the Hindu-Arabic number symbol system, the recursive structure starts showing at 10. When a child grasps the recursivity of the number symbol system, she understands that the same system can be used to denote larger and larger numbers. Usually at this stage of grasping the recursive character of the number system, children realize that the same operations work for numbers however large they become, and they often also develop an early understanding of the concept of infinity (Tirosh, 1999; Monaghan, 2001). However, a notion of infinity is not necessary for a system to count as arithmetical. The Mayans, for example, had great ability with calculations of natural numbers, and thus, we should understand their system as arithmetical. Yet they did not seem to develop an explicit notion of infinity (Ifrah, 1998).

To sum up, “arithmetic” refers to recursive verbal or symbolic systems with sufficiently extensive linear structure of discrete quantities, as well as operations (e.g., addition, multiplication) on them. Any treatment of quantities developmentally prior to that should be called *proto-arithmetic.* The term “natural numbers” (or just “numbers”) refers to the objects of arithmetic. The quantity concepts used to describe proto-arithmetical ability should be called *numerosities*^2^. Under this definition, the infant and animal abilities refer to numerosities and are thus proto-arithmetical, but that is also the case with cultures who have only limited systems of numeral words or number symbols. With these conceptual clarifications in place, we can now get an explicit theoretical framework in which to study the development of arithmetical knowledge.

## From Proto-Arithmetic To Arithmetic

### Proto-Arithmetical Ability

In the research of numerical cognition, it is standardly accepted that the acquisition of arithmetical knowledge is a cognitive process that is dependent on the proto-arithmetical abilities (see, e.g., Dehaene, 1997/2011; Spelke, 2000; Carey, 2009). I will discuss the justification of this assumption later, but for now let us accept the consensus view and see what it implies. With the conceptual clarifications presented in the previous section, we can identify the key stages in the acquisition of arithmetical knowledge and skills. The first stage is the acquisition of the proto-arithmetical abilities, which includes the core cognitive abilities but is not limited to them. The second key stage is moving from proto-arithmetical processing of numerosities to proper arithmetical cognition. Crucially, this requires acquiring the knowledge that natural numbers form a discrete and linear progression that continues indefinitely, often called either the *exact number system* (e.g., Izard et al., 2008; Castronovo and Göbel, 2012) or the *discrete number system* (e.g., Cantrell and Smith, 2013; Menary, 2015) in the literature. Finally, at the third key stage, we can acquire a formal understanding of arithmetic and treat arithmetic as a subject of mathematics in its generality, proving theorems about all natural numbers. The move from the second stage (arithmetical knowledge) to the third stage (what I call *formal* arithmetical knowledge) is an important topic, as it distinguishes between the ability to carry out arithmetical operations and understanding formal arithmetical structures. We will return to this topic in the final section of this paper, but for now our main interest concerns the move from proto-arithmetic to arithmetic.

Although the details are still a matter of debate, it is generally accepted that human infants and many nonhuman animals process observations of objects and organisms in their environment in terms of quantities. This ability is thought to be a genetically determined adaptation and universal to humans (Dehaene, 1997/2011). It is not known whether the ability is present at birth, so a common characterization of it as *innate* is somewhat problematic, as are indeed many uses of that word for human cognitive capacities (Bateson, 1991; Griffiths et al., 2009). It should also be noted that like with many abilities described as universal, due to the possibility of developmental dysfunctions, we cannot assume the quantitative ability to be present in every individual. While such details are highly important for many purposes, the important point in the present context is that at least part of the proto-arithmetical ability is present early in the ontogeny and is thus thought to emerge independently of linguistic or other culturally dependent instruction.

According to the most commonly accepted theory, this non-symbolic treatment of quantities is based on the so-called *cognitive core systems* (Spelke, 2000). In the literature, two such systems have been identified. First of these is a system for parallel individuation (PIS) (or object tracking, OTS) that allows determining the amount of objects in the field of vision without counting (Starkey and Cooper, 1980; Spelke, 2000). This ability is called *subitizing*. The second system allows estimating the numerosity of a group of objects and determining differences in group sizes. It is referred to as either the *approximate number system* (ANS), the analogue magnitude system, or number sense (Dehaene, 1997/2011). Both abilities are used to treat numerosities, but they have important limitations, which make them proto-arithmetical rather than arithmetical. Subitizing deals with discrete numerosities, but it only works for small quantities, usually up to three or four objects. The ANS works for larger groups, but it is an estimation system that becomes increasingly inaccurate as the collections become larger, and is thus generally not considered to be discrete.

Based on many experiments, it is commonplace to accept that subitizing and ANS-based estimating are indeed two separate abilities for treating quantities, arising from separate cognitive core systems (Feigenson et al., 2004; Agrillo, 2015)^3^. Some scientists postulate that the ANS is key to the development of number concepts and arithmetic (e.g. Dehaene, 1997/2011; Halberda and Feigenson, 2008), while others see subitizing and parallel individuation as the prevalent core cognitive abilities in that development (e.g., Izard et al., 2008; Sarnecka and Carey, 2008; Carey, 2009; Carey et al., 2017; Cheung and Le Corre, 2018). In my previous work (Pantsar, 2014, 2015, 2016, 2018), I have argued that both abilities can be considered to be proto-arithmetical, i.e., there is a relevant sense in which both abilities contribute to the development of arithmetical cognition. Further empirical evidence of this can be found in, e.g., VanMarle et al. (2018), whose experiments imply that measures of both the PIS and the ANS predict knowledge levels of numbers. For the present purpose, however, the particular theory of how proto-arithmetical abilities develop from cognitive core systems is not crucial. The important part is to establish that there indeed are proto-arithmetical abilities that are not culturally determined.

The evidence for this is strong. It is perhaps not surprising that our close evolutionary relatives, such as primates, show similar proto-arithmetical ability as humans (Hauser et al., 2000). More surprising is the finding that the abilities are remarkably similar also in rats, birds, and even fish. For example, robins in New Zealand have shown that they can discriminate between one, two, three, and four objects, but after four they can only consistently distinguish quantities when the ratio is at least 1:2 (Hunt et al., 2008). The remarkable similarity in the results in infant and animal studies suggests that both the subitizing ability and the ANS are either comparatively early development in the evolutionary genealogy of animal species or such useful cognitive mechanisms that they have developed several times independently. However, currently this question concerning the animal and early human ability with numerosities can only be speculated on. We simply do not know for sure when and how the core cognitive proto-arithmetical ability has developed during phylogeny.

Neither do we know for what purpose it developed, although there are plausible hypotheses for this. Being able to estimate the size of collections is clearly a useful ability in many situations. Going for the larger pile of food and many similar cases, however, can be explained without evoking any ability to distinguish between *discrete* quantities. So why can many animals also exactly discriminate between small numerosities? In nature, there are many situations where it is indeed the discrete quantity – rather than, say, the total visible size – which is important. If an animal is able to distinguish between three and four predators, this ability to establish the quantity of the animals is likely to be more useful than the mere ability to determine the total size that it has observed. After all, four foxes pose a different threat than one wolf, even if the total observed size (e.g., visible surface area) was the same. Similarly, keeping track of one’s offspring, or being able to determine the right hole in a burrow, for example, provide clear cases where rough estimation is an inferior strategy compared to discriminating the discrete quantity of objects or other living beings.

Regardless of the details of such explanations, however, we can be confident that there is a level of proto-arithmetical treatment of quantities also in human cognizers that is genetically determined and not culturally learned. In this paper, I want to study the question what the conditions are that allow developing the proto-arithmetical abilities into arithmetic proper, starting from the core cognitive quantitative ability. While we appear to share much of the primitive systems for treating quantities with many nonhuman animals, somehow humans (although not all humans) use these foundations to develop arithmetic. This is often seen as one of the most important challenges in explaining the move from proto-arithmetic to arithmetic (see, e.g., Brannon and Park, 2015): What are the key cognitive and conceptual leaps that we make in turning the primitive ability into something exact and conceptually coherent?

### Developing Arithmetic

The conceptual leaps involved in developing numerical cognition beyond core cognitive abilities are particularly interesting because they are not universal to all humans and not even totally exclusive to humans. While animals generally possess only primitive proto-arithmetical ability, through extensive training, animals such as chimpanzees and parrots have shown that they can learn numerical skills, such as simple addition, beyond those given by the core cognitive systems (by first learning human language labels for numerosities; see, e.g., Pepperberg, 2012). On the other hand, there are human cultures, such as the Amazonian tribes of Pirahã and Munduruku, whose skills with quantities have not developed considerably beyond the primitive systems shared with many nonhuman animals (Gordon, 2004; Pica et al., 2004; Dehaene et al., 2008). It seems thus likely that there are important cultural factors involved in developing and refining the primitive abilities toward an exact treatment of quantities. Whereas some level of proto-arithmetical ability is universal, arithmetical cognition is culturally specific. Thus, having proto-arithmetical ability is clearly not a sufficient condition for developing arithmetic. Indeed, while the Pirahã and the Munduruku give us much discussed examples of non-arithmetical cultures because they do not have even rudimentary numeral systems in their languages, the development of arithmetical systems has been quite a rare occurrence in the history of mankind.

Neither can we categorically state that the proto-arithmetical ability is a *necessary* condition for developing arithmetical cognition. There are at least two feasible scenarios in which arithmetical cognition can develop without recourse to the proto-arithmetical ability. First is the case of artificial intelligence. Although the technological details may turn out to be highly difficult, it is possible to simulate human arithmetical ability with computer programs. Yet there would seem to be little motivation for programming computers to first treat quantities in a proto-arithmetical manner in order to simulate the development of human arithmetical ability. More interesting than this scenario, however, is the possibility that a human cognizer could learn arithmetic without employing the cognitive scaffolding provided by the proto-arithmetical ability. While the evidence strongly suggests all human beings have genetically determined proto-arithmetical ability to treat quantities, it does not necessarily follow that all humans actually *use* that ability in learning arithmetic. Therefore, we should also consider the possibility that one can learn arithmetic in a manner that is conceptually independent of the proto-arithmetical ability. Indeed, without further evidence, we cannot rule out the possibility that this is actually how *most* – or perhaps even all – humans learn arithmetic.

While we cannot dismiss this possible discontinuity between proto-arithmetic and arithmetic, there is empirical evidence against this happening regularly. We cannot currently conclusively establish the role that proto-arithmetic plays in the development of arithmetical cognition, but there is a lot of data suggesting at least a strong correlation between the two. Nieder et al. (2006), for example, conducted an experiment in which monkeys were presented objects one by one to simulate a non-verbal account of counting. As expected, the activated brain areas were partly different than when the objects were presented all at once, i.e., when only the subitizing and ANS-based estimation abilities were used. However, the experiment revealed that a large part of the activated neurons were in fact the same in the two settings, suggesting that when counting, the monkeys were dealing with (partly) the same representations for numerosities as when subitizing or estimating with ANS. The experiment was conducted on monkeys, which could make its applicability to human cognition questionable, but Piazza et al. (2007) have shown that when observing numerosities, the same areas of brain (in the intraparietal cortex) are activated in humans as in monkeys. Indeed, Cantlon and Brannon (2006) have shown that college students show similar patterns to monkeys and rats when it comes to solving number-ordering and quantity estimation tasks. Finally, research on non-arithmetical tribes shows that education in verbal numerical processing enhances the acuity of the ANS, thus showing that the connection between linguistic numbers systems and the core cognitive ability also goes the other way (Piazza et al., 2013).

Of course none of this evidence manages to show unassailably that humans need to use their core cognitive abilities for treating quantities when learning arithmetic. There remains at least a theoretical possibility that the systems are not connected. What the data do suggest quite strongly, however, is that such developmental independence from the proto-arithmetical origins is unlikely. The hypothesis that gets most support from the empirical studies is that the connections between core cognitive proto-arithmetical and arithmetical ability are due to the latter ability developing (at least partly) on the basis of the former (e.g., Dehaene, 1997/2011; Butterworth, 1999; Feigenson et al., 2004; Izard et al., 2008; Sarnecka and Carey, 2008; Carey, 2009; Nieder and Dehaene, 2009; Brannon and Merritt, 2011; Spelke, 2011; Castronovo and Göbel, 2012; Piazza et al., 2013; Nieder, 2016, 2017; Carey et al., 2017; Núñez, 2017). On an organizational level of the brain, this hypothesis makes sense. If there is an existing system in place for treating quantities, due to considerations of metabolic costs and efficiency, we should expect new quantitative knowledge to develop in connection to this system, rather than starting from scratch.

### Language

Based on the above considerations, it is unlikely that humans generally learn arithmetic independently of the core cognitive proto-arithmetical abilities. But what are the factors that need to be present in that development? The first obvious requirement is that the language must have enough expressive power for arithmetic. The Pirahã numeral system, for example, which includes words roughly only for “one,” “two,” and “many” (Gordon, 2004), is clearly detrimental for the development of arithmetic^4^.

That, however, is only the most extreme example of a language that does not allow for the development of arithmetic. Indeed, it would appear that any language that does not have a recursive numeral system is detrimental to arithmetical cognition, since the meaning of each numeral has to be grasped separately. When it comes to human languages in general, Hauser et al. (2002) have argued that recursion, i.e., the lack of upper bounds in the length of syntactical constructions, is a (even *the*) main characteristic. Yet, as Everett (2005) has argued, it is not clear that the Pirahã language, for example, has this characteristic. It is natural to hypothesize that this connection between the putative non-recursive character of the Pirahã language and their lack of arithmetical ability is not coincidental.

However, it is not obvious that such a hypothesis is warranted. While many researchers (e.g., Bloom, 2000; Maddy, 2014) hold that the recursive structure of our language is indeed key to grasping the natural number structure, this is not universally accepted. Gelman and Butterworth (2005), for example, have argued for the position that we can have non-linguistic representations of natural numbers. Their argument is based on the Oksapmin people of New Guinea, whose indigenous language does not have number words, but rather use body positions to signify different quantities. However, when they entered plantations to work, the Oksapmin were quick to learn counting rules and the numeral vocabulary. Gelman and Butterworth argue that this could not have been possible without a prior non-linguistic representation of natural numbers. Obviously, there are limits to how far such non-linguistic representation of quantities can go, but the existence of an even somewhat extensive collection of non-linguistic representations provides interesting questions about the role of language in grasping the concept of natural number. Indeed, could we plausibly have a pre-linguistic conception of exact quantity that goes beyond the proto-arithmetical origins?

This hypothesis may be possible to make on the basis of the Oksapmin ability, but we can also understand the Oksapmin ability with quantities – if their learning curve indeed is exceptional – by other means. Gestures with body parts have often been seen as a crucial part in learning language (see, e.g., Goldin-Meadow and Alibali, 2013). While the validity of this hypothesis remains to be decided, there have been important results connecting gestures and language. Xu et al. (2009), for example, showed that symbolic gestures and (spoken) language employ a common neural system. With such results, it is perhaps more relevant to consider the position that takes language as a broader concept that includes sensory motor aspects (Hauser et al., 2002). Thus, under the broad concept, the Oksapmin ability would not count as non-linguistic representation of quantities, as the body part for, say, “six” may in fact function much like a numeral word or a number symbol.

However, it is not easy to make the choice between a broad and a narrow conception of language in any general manner. When it comes to the importance of language for the development of arithmetic, it would seem to be quite useful to focus on the narrow conception of language that includes only verbal representations of quantities – or perhaps an even narrower conception that only includes languages that have recursive numeral systems. As defined in this paper, arithmetic requires a sufficiently rich discrete linear system of numerals or number symbols. This might be seen as implying that arithmetical cultures require languages that have recursive numeral systems. Yet arithmetic can also be practiced by a simple tallying (stroke notation) system in which the number of strokes determines the quantity. A culture can have such a system without having a recursive numeral system in their language. Thus, while undoubtedly beneficial, having a recursive numeral system in their natural language is not a necessary condition for developing arithmetic^5^.

The above considerations suggest that we should not be too hasty in making connections between the characteristics of the language of a culture and its conduciveness for developing arithmetic. Indeed, we should also refrain from making a choice between the narrow and wide conceptions of language. For different stages in the development of arithmetical cognition, we can employ different conceptions of language. On the one hand, we can be interested in verbal languages when studying, for example, the way numeral systems influence our cognitive processes. On the other hand, when examining the first ways of representing and communicating quantities, we should not necessarily dismiss systems of body positions as non-linguistic. Such a system can have distinct representations for a considerably large amount of quantities as well as allow operations (such as addition) on them. However, there is a limited range of intelligible gestures so expressing large numbers becomes cumbersome, time-consuming, and error-prone. It is therefore clear that verbal and symbolic numeral systems generally carry an advantage over body position systems. But this advantage is not always manifested. The body position system of the Oksapmin, for example, is richer than the verbal system of the Pirahã.

It may not always be clear what conclusions one should draw from the above considerations, but it becomes evident that the role of linguistic systems in the development of arithmetical cognition provides us with many difficult questions. It has been established, for example, that many Western children 3–5 years of age – who are familiar with number words – are more successful in referring to small quantities with hand gestures rather than words (Gunderson et al., 2015). This suggests that manual representations such as extending fingers can be the primary way of representing quantities, and it can take children longer to grasp the linguistic connection between the number words and quantities. Such a hypothesis also gets support from the data indicating that after children first learn the sequence of numerals (starting from the age of two), it takes them some time (usually until the age of four) before they are able to match the number words successfully to quantities (Fuson, 1988; Wynn, 1990; Davidson et al., 2012). Overall, it appears that learning the necessary expressions and linguistic content to represent quantities is not sufficient. In addition, children need to learn the so-called *cardinality principle* (Wynn, 1990; Sarnecka and Carey, 2008), i.e., the knowledge that in the counting process the last uttered numeral signifies the quantity of the collection. This is often seen as a key stage in children’s general learning that each numeral refers to one, and only one, exact discrete quantity (Sarnecka and Carey, 2008; Carey, 2009; Beck, 2017).

It is undoubtedly the case that the characteristics of a particular language can be beneficial or detrimental for developing numeral systems suitable for arithmetical cognition. The recursivity of our contemporary numeral systems clearly provides a better platform for developing arithmetic than one that does not show any recursive structure. However, it is possible that numeral systems that show recursivity were the *consequence* of rather than the *cause* for developing arithmetic. It could be that using primitive symbolic systems of treating quantities, such as tallying, necessitated developing a recursive numeral system. In this case, as well as studying the effect of languages on arithmetical cognition, we should also look for other cultural factors that may have influenced the path our treatment of quantities has taken. Perhaps the Oksapmin, for example, have had some extra-linguistic cultural reason which made it more useful to keep track of and communicate quantities – some reason that the Pirahã did not have.

### Beyond Language

The above considerations bring us to the basic problem when explaining the development of arithmetical cognition. If we study, as I have argued we should, arithmetic as a wider phenomenon than our contemporary systems, we face an interesting situation. Unlike the alphabet, for example, which is often thought to have developed only once during the course of known human history (see, e.g., Sampson, 1985), arithmetic is known to have developed several times independently (Ifrah, 1998). While the particular systems of arithmetic have had different characteristics, they also show great similarity in both counting and operations like addition and multiplication. For almost all children growing up in arithmetical cultures, basic arithmetic is easy to learn. Most known indigenous languages do have some kind of a numeral system, and many of these systems show recursivity in some numeral base (Ifrah, 1998). It is also often recognized in the modern literature how important the development of number systems can be for people. Everett, for example, argues that “spoken numbers and written numerals were pivotal to radical transformations in a variety of cultures millennia ago” (Everett, 2017, 238).

Yet at the same time, unlike language, arithmetic even in early forms is far from being a universal ability. Most cultures have not developed anything resembling arithmetic as characterized in section “What is Arithmetic?” Why is this the case? If the universal abilities of subitizing and the ANS determine (at least partly) the content of our arithmetic, how is it possible that so many cultures have not made the step from proto-arithmetic to arithmetic that seems so obvious to us? The numerically limited languages of Munduruku and Pirahã are in this sense only the tip of the iceberg. They have received a lot of attention due to their lack of numerals beyond the first few, but in this they are exceptions (Everett, 2017). Most cultures do have some kind of a numeral system, yet it is often not developed systematically into arithmetic. The numerals may only reach four, ten, or some other relatively small numeral, and even simple operations like addition are not used.

This implies an intriguing mismatch. On the one hand, numerical systems and their applications are seen as great advantages for a culture, causing “radical transformations.” On the other hand, many cultures have not developed even basic arithmetic, and some do not even have numeral words. Looking at the great facility with which young children in arithmetical cultures learn to count and calculate, it seems quite odd that cultures can exist for thousands of years without referring systematically to quantities larger than two, let alone grasping the general idea that natural numbers form a progression that goes on and on. Perhaps not grasping the abstract idea of infinity, or the explicit idea of an indefinitely continuing succession, is understandable, but not using discrete quantities for objects in their environment seems to have clear practical disadvantages.

This mismatch requires us to reconsider the importance of arithmetic. Because of the very fact that many cultures have not innovated arithmetic, we should be careful in making general claims about the practical conditions and advantages of developing arithmetical systems. Many aspects of modern Western arithmetic, such as the focus on proofs concerning all numbers, are not necessarily the kind of practical advantages that motivate developing a system of discrete numbers and their operations. Instead, it is likely that the motivating factors have had direct applications, which are facilitated by the ability to understand and communicate discrete quantities. Traditionally, at least four such factors for mathematics have been emphasized in the literature: agriculture, trade (and other commerce), navigation, and astronomy/astrology (Boyer, 1991). It is understandable to presume that differences in the importance of these pursuits have a lot of influence on how mathematics develops. Trade, for example, would usually be something in which developing arithmetic proves useful, whereas focus on navigation, for example, could explain preference for developing geometry^6^.

The different circumstances may also give us insight into the question why people like the Pirahã and the Munduruku have developed such limited systems and applications for numerosities. There is nothing to suggest that they have some innate cognitive disadvantage in developing mathematics. It could simply be that in their cultures there was not the same kind of incentive to develop a numeral system, and thus, people growing up in those cultures were not offered the kind of cognitive tools that enable children in other cultures to learn arithmetic^7^. For example, even though the Pirahã do practice trade, no record is kept about it. As hunter-gatherers, they do not practice agriculture or store food (Everett, 2017).

Considering the vastly different cultural practices, it becomes less surprising that not all cultures have developed extensive numeral systems, let alone arithmetic. To further elucidate the vast difference between the Pirahã and modern Western cultures, it is interesting to note that the Pirahã also cannot draw even familiar shapes like trees, animals, or people (Gordon, 2004). On the other hand, the Pirahã have sophisticated knowledge and skills in acquiring food in a difficult environment (Everett, 2017).

This way, it is easy to be blind to the innovativeness of ideas that are familiar in one’s own culture. There have been intellectually highly developed cultures, such as the Incas, who never invented the wheel. The Incas would most probably have found the invention useful, but for some reason it never occurred to them, nor did they communicate with other cultures who had developed the wheel. Similarly, extensive numeral systems and arithmetic would have been useful also for many cultures that never developed them. For some reason, either the creative process necessary for extensive numeral systems did not occur, or the numeral systems failed to establish a sufficiently important status in the particular cultures.

Considering these two options, there is an interesting potential difference when it comes to inventions such as the wheel and numeral systems. The wide existence of numeral systems in indigenous languages all over the world is sometimes seen as evidence that they were an early feature of human languages (see, e.g., Everett, 2017). This is hard to establish, since the competing hypothesis that numerals were a late invention also gets support, for instance, from the great variety that the numeral systems have (Ifrah, 1998). But for the sake of the argument, let us accept that numerals were present early on in human languages. This would suggest that ancestors of cultures like Pirahã and Munduruku (both relatively young cultures on the scale of the *Homo sapiens* era) at some point in fact had numerals in their languages, but for some reason they later disappeared. In this case, the usefulness of numerals must have been limited. This is impossible to confirm, but it becomes quite understandable based on the above considerations that the abilities that come so easily even to young children in many cultures are in fact made possible by the specific circumstances and practices in these cultures, which are due to a particular line of cultural heritage of ideas and artifacts (Henrich, 2015). Any discussion on cognitive abilities must take these factors into account. The possession of the ability to add, for example, has been made possible by a wide range of cultural and practical factors, which have enabled the development and communication of the necessary conceptual tools, as well as providing the motivation and opportunities to develop the ability. Thus, the move from proto-arithmetic to arithmetic requires studying the question in a perspective that takes into account the cultural influences in all their varieties.

At the same time, there is also much about arithmetical cognition that is constant in different cultures. Not only have different cultures developed equivalent systems of numbers and their operations, but as we will see in the next section, the same areas of the brain are used in arithmetical cognition. While the development of arithmetical knowledge and skills seems to be in important ways determined by our cultural circumstances, as seen in section “Developing Arithmetic,” there are extensive data suggesting that it is based on core cognitive abilities that are culture-independent. Thus, the challenge becomes assimilating these two approaches, i.e., explaining the development of arithmetic in a way that is based on its core cognitive origins, but also includes the culturally dependent aspects. In the first two sections, we have established the necessary conceptual distinctions in explaining the cognitive basis of arithmetical knowledge. We have also seen that cultural factors play a key role in determining how abilities with quantities develop. In the rest of this paper, we will study a framework that has been proposed as a solution to the problem of combining these aspects into an empirically and philosophically plausible theory.

## Enculturation as the Answer?

### Enculturation and Neuronal Recycling

Based on the great cultural variation in numerical ability, it is clear that all ontogenetic and phylogenetic accounts of the development of arithmetical knowledge need to look beyond the cognitive core systems. The considerations in the last section show that linguistic factors play an important role both in the phylogeny and ontogeny, but a comprehensive explanation must include cultural aspects also beyond them. Thus in the philosophy of mathematics, in addition to accounts of phylogeny – to which we return to in the next subsection – we should look for frameworks of ontogenetic development that allow for a multitude of cultural influences. That fits well with the framework of Menary (2015) in which he presents mathematical cognition as a case of *enculturation*. Enculturation refers to the transformative process in which interactions with the surrounding culture determine the way cognitive practices develop (Menary, 2015; Fabry, 2018a). Through the mechanism Menary (2014) calls “learning driven plasticity,” new cognitive capacities can be acquired due to the neural plasticity of the human brain, which allows for both structural and functional changes (Dehaene, 2009; Ansari, 2012; Anderson, 2015).

This setting is important for the philosophy of mathematics as it distinguishes Menary’s position from conventionalist philosophy of mathematics (e.g., Wittgenstein, 1956/1978; Field, 1980). Whereas the stronger conventionalist position holds our cultural influences to be either entirely or at least mostly responsible for the development of mathematics, Menary’s (2007) model assumes that mathematics both in phylogeny and ontogeny is made possible by the *cognitive integration* of “multiple cognitive layers where neural, bodily, and environmental processes all conspire to complete cognitive tasks” (Menary, 2015, 2). Following the niche constructionist theory (e.g., Laland et al., 2000), Menary argues that the phylogeny of human cognition develops by “active embodiment in a socially constructed niche” (Menary, 2015, 3). In the ontogenetic development, children develop their skills and knowledge in the shared cognitive niche, which includes representation systems, tools, and practices (Clark, 2006; Stotz, 2010; Menary, 2014). Menary’s framework is therefore not social constructivism in a radical, conventionalist sense. Rather, his position is characterized as the dynamics of integrated cognitive systems being “jointly orchestrated by biological and cultural functions” (Menary, 2015, 3).

Therefore, Menary’s framework of enculturation appears to be a good fit with the considerations in the previous two sections. If we accept that the proto-arithmetical core cognitive ability (partly) determines the developmental path of arithmetic, our theoretical framework must include biological functions as both ontogenetically and phylogenetically active components in the formation of arithmetical cognition. But we have also established that the core cognitive ability underdetermines the content of arithmetic, and in many cultures, it is not developed into proper arithmetic at all. Thus, the cultural functions in the formation of arithmetical cognition must be included in a feasible theoretical framework. In the present context, the first key insight into Menary’s argumentation is that when it comes to abstract symbolic thought – like arithmetic – cognitive integration does not take our cognitive abilities to be innate. This is based on a sound evolutionary argument. On an evolutionary scale, there simply has not been enough time for arithmetic to have had enough impact on the structure and function of the brain. While it is widely accepted that arithmetic is partly based on an innate proto-arithmetical ability with numerosities, this can only explain part of the development of arithmetical cognition. Instead of there being some kind of innate functional disposition for arithmetical ability, it is the plasticity of our brain that plays a key role: old neural circuits are redeployed to new functions in a process called *neuronal recycling* (Dehaene, 2009; Menary, 2014).

This redeployment is a specification of what Anderson calls *neural reuse* (Anderson, 2010, 2015; Fabry, 2018a; Jones 2018). Neural reuse refers to the general process of “circuits (continuing) to acquire new uses after an initial or original function is established” (Anderson, 2010, 245). The neuronally recycled functions, Menary (2014) argues, are culturally specific. Although its details can differ considerably based on the particular formulation, the theory of neuronal recycling provides a feasible platform for explaining how enculturated mathematical knowledge can manifest itself in the brain. In addition to the theoretical suitability, neural reuse can be plausibly used to explain many empirical data (Anderson, 2015, 2016). Research on nonhuman primates shows conclusively that neural reuse happens on the level of single neurons. Work with animals with simple neuronal systems (such as the soil-dwelling roundworm *Caenorhabditis elegans*) shows that the synaptic structure between individuals can be largely identical, yet there can be vast functional differences in the neurons, to the point that they are even associated with completely opposite tasks (Cisek and Kalaska, 2010; Varshney et al., 2011; Anderson, 2015).

Importantly, together with the proto-arithmetical processing of quantities, enculturation and neuronal recycling can also explain why there is little inter-personal variance in the brain regions that are employed for mathematical processing. Butterworth (1999) has argued – based on the existence of core cognitive numerical abilities – that there is an innate cognitive module for treating numerosities. His argument is set in the framework of Fodor’s (1983) theory of the modularity of the mind. Butterworth focuses on one particular aspect in Fodor’s theory: the distinction between functionally specific input modules and non-modular central processes. In Fodor’s characterization, “input systems function to get information into the central processors” (Fodor, 1983, 42). Butterworth describes central processes as such that “we can choose whether to operate them or not; we are not born with brain circuits specialized to do them; and they need learning” (Butterworth, 1999, 5). Furthermore, he contends that Fodor (1983) is committed to arithmetic being such as central process. But since empirical data show that numerical ability develops on the basis of the input module for processing numerosities, Butterworth argues that arithmetic cannot be a central process. Since there are in fact brain circuits specialized for treating numerosities, the argument goes, every brain is “hardwired” for arithmetic.

However, this line of argumentation is questionable. At first, arithmetical ability might seem to be an empirically good fit with the modularity theory, since data show little inter-individual variation in the brain regions – belonging to the parietal, frontal, and temporal lobes, in particular the intraparietal sulci – used in numerical processing (Dehaene, 1997/2011; Ansari, 2008). This inter-individual consistency in the brain regions may seem to support the idea of functional modularity, which could be seen as being in conflict with the theory of neuronal recycling. But the assumption of modularity is problematic when we keep in mind that arithmetical cognition is much too late a phylogenetic phenomenon to have feasibly caused the development of a functionally specific module in the mind (Fabry, 2019). The evolutionary emergence of an “arithmetic module” would take periods on a totally different time-scale from the approximately 5,000 years ago that first symbolic number systems have been found (Schmandt-Besserat, 1996).

Therefore, we should look for other answers than modularity for the small inter-individual variation in the brain regions used for arithmetical cognition. Fortunately, enculturation framework can provide such an answer, once it is recognized that mathematical ability is not purely culturally determined. For this reason, it is crucial for the enculturation and neuronal recycling framework that we can establish that arithmetic indeed develops on the basis of the proto-arithmetical ability. With this connection, it is to be expected that the neural circuits used for arithmetic are similar between individuals, given that there is a genetically determined propensity to use certain brain areas for the processing of quantitative information. This falls under what Anderson (2015) calls the *functional bias* of brain areas. Indeed, there is solid empirical evidence that this is the case. The intraparietal sulcus, for example, is used for both the core cognitive and arithmetical treatment of quantities (Dehaene and Cohen, 2007). One empirically supported hypothesis that helps explain the lack of variation in the brain areas used for arithmetical processing is that already on the core cognitive level particular neurons are associated with numerosities. Research shows that distinct groups of neurons in the parietal and frontal lobes appear to be associated with different numerosities. The firing of these neurons is independent of modality so the same group activates regardless of whether we see two objects or hear two tones (Nieder et al., 2002; Nieder and Miller, 2003; Nieder and Dehaene, 2009; Nieder, 2012, 2013, 2016). Studies also show that there is a two-way connection between our proto-arithmetical ability and acuity with number concepts. For example, higher mathematical skills correlate with better performance in estimation tasks (Cantlon and Brannon 2006; Brannon and Merritt, 2011). Data also show that developmental dyscalculia and damage to the proto-arithmetically important areas in the brain are connected to lower mathematical skill levels (Dehaene, 1999; Butterworth, 2010).

Thus, the theory of enculturation based on neuronal recycling fits well with the research on proto-arithmetical treatment of quantities. Brain areas for numerical treatment are not established randomly. Rather, the existing ability for quantitative processing of observations determines (among other, such as language-related, factors) which neural circuits are used in the enculturated learning of mathematical concepts. The brain regions used for numerical processing change – for example, as exact number concepts are acquired, the neural activity moves increasingly from the right intraparietal sulcus to the left one – but this change is generally similar between individuals (Emerson and Cantlon, 2015).^8^ In this field, rapid progress is being made, and hopefully in the near future, we will have a much better understanding of the different cognitive processes in the development of arithmetical cognition. From what has already been established, however, enculturation and neuronal recycling seem to get solid support from the empirical data and provide a plausible mechanism for the emergence of arithmetic. It is only in this way – combining the core cognitive systems and neuronal recycling – that the idea that brain is “hardwired” for arithmetic makes sense. Any stronger conception of a hardwired numerosity module is unsupported by evidence.

### Cultural Evolution

As important as the above considerations on the neuronal basis of arithmetic are, they only form one part of explaining the emergence of enculturated arithmetical knowledge. What needs to be clarified next is how arithmetic developed into its current state. As was argued in section “From Proto-Arithmetic to Arithmetic,” it is easy to accept that having an extensive system of discrete quantities, for example, is beneficiary for many practical purposes, such as trade and agriculture. Yet it is by no means obvious how cultures have managed to develop their knowledge and skills with numbers from the modest core cognitive origins to the modern discipline. Neuronal recycling provides a plausible mechanism to explain how enculturation works on the level of the brain. But in order to make sense of the phylogeny and history of arithmetic, we also need to identify how enculturation works on the level of cultural practices. How and why do some cultural practices endure and develop, while others are forgotten?

This question is particularly interesting in the case of mathematics because of the immense cultural differences, including cultures with little or no mathematics. As was argued in section “Beyond Language,” one potential explanation for not developing mathematics is the general lack of applications for mathematical knowledge. Alternatively, it is possible that in some cultures advancements in mathematics (or toward mathematics) were for some reason not widely learned and may have been consequently lost, perhaps because the culture divided into separate populations^9^. But in cultures where mathematics exists, it is clearly the product of a long development. This is consistent with Henrich’s (2015) theory of *cumulative cultural evolution* as the way human cultures develop their knowledge and skill sets. Helpful inventions are improved upon in small generational increments, and in large enough societies – or ones with extensive interactions with other societies – this process can establish a status of knowledge and skills where it is no longer tied to a small group of individuals. Arithmetic, for example, developed into such a skill in many cultures. It is taught to young children with a highly developed methodology, thus facilitating the learning process for each individual.

Cumulative cultural evolution is a trans-generational process that can help explain how cultural transmission happens in the enculturation account. We can thus construct a biologically and culturally determined model of the development of arithmetical knowledge in the enculturation account. Arithmetical ability is partly determined by the core cognitive ability with quantities that we already possess as infants. Due to cultural transmission, in most cultures, this ability is extended to include a system of numerals to refer to small discrete numerosities. At this – still proto-arithmetical – level, people are able to keep track of small quantities and have applications for them. On some occasions, cultures develop their numeral systems to have a recursive base, which allows for the unrestricted construction of new numerals as well as extending the operations for this domain. At this stage, we can speak of arithmetical knowledge and skills. Arithmetic can then be developed to include general proofs, and it can be presented as axiomatic systems in formal languages. It is important to note that throughout this development, having the suitable linguistic tools is crucial to reaching new stages in arithmetical knowledge, consistent with my treatment of the topic in section “Language.” But equally importantly, there are also factors beyond language, making the framework compatible with a multitude of cultural factors, as specified in section “Beyond Language.”

If the above theory of the enculturated character of arithmetical knowledge is accepted, what should we expect from the development of arithmetic in different cultures? There are at least three characteristics we would expect to be present. First, if the hypothesis of enculturated arithmetic were true, we would not expect arithmetical ability to be universal and essentially unchanged between cultures. If it were, it is possible that enculturation is not essential to the development of arithmetical cognition. There would likely be enculturated aspects, as well – concerning at least notation and practice – but Menary’s (2015) argument is that enculturation is a more robust, an essential characteristic of arithmetical cognition.

The second expected characteristic is that, while not always actualized, the *potential* for arithmetical cognition would need to be universal. An important part of the enculturation theory is that arithmetical cognition is possible by redeploying neural circuits evolutionarily developed originally for different purposes. Arithmetic is too young a development to be the product of biological evolution, but the potential for redeploying the necessary areas of the brain has to be present universally in humans.

Finally, a third expected characteristic of the enculturation account is that we should expect to see a certain degree of variance in the arithmetical systems that different cultures have developed. If all arithmetical cultures developed the theory of natural number essentially similarly, it could count as evidence against the enculturation thesis. In such a case, the enculturated aspects would appear to be of minor importance, whereas the essence of arithmetic would be better seen as something universal, culture-independent – perhaps determined by the proto-arithmetical core cognitive ability.

The first expected characteristic – arithmetic not being universal – is clearly fulfilled, as there are several non-arithmetical cultures. The Pirahã and the Munduruku are the most famous cases in the literature, but there is still a number of other cultures without developed arithmetic, for example, in Papua New Guinea (see, e.g., Matang and Owens, 2014). As it becomes increasingly difficult to live in isolation from wider cultural influences, this is quickly changing. Historically, however, the development of arithmetic is much more an exception rather than the rule. From the vast amount of cultures in the pre-Columbian Americas, for example, in addition to the well-known Olmec-Mayan arithmetic, to the best of our knowledge, only few other cultures developed even rudimentary ability with numbers (Everett, 2017). There are many explanations for this, ranging from the lack of any kind of symbolic language to the fact that the vast majority of pre-Columbian cultures were hunter-gatherers. Since the most popular hypothesis currently is that the Native American people came from a relatively small group of ancestors, the differences are unlikely to be due to any genetic reasons. Thus, the importance of culture for the development of numerical thinking is evident.

There is equally strong evidence for the second expected characteristic, the universal potential for arithmetical cognition. As we have seen, the core cognitive abilities with numerosities are universal. Even if one rejects the hypothesis of the proto-arithmetical core cognitive abilities being (at least a partial) foundation for the development of arithmetical cognition, the evidence for the universal *potential* remains strong. It is obvious that people from a wide variety of ethnic and cultural backgrounds learn arithmetic. In this, people like the Pirahã are the exceptions resulting from the kind of isolation that most modern cultures do not live in. The vast majority of people in the Americas nowadays learn at least basic arithmetic regardless of their ethnic background. This is of course not peculiar to the Americas as, to the best of my knowledge, there are no reports of people lacking the cognitive abilities to learn arithmetic due to some genetic reason.

There is very strong evidence also supporting the third characteristic, the expected cultural variation. The Mayans, for example, had an arithmetical system that allowed calculation with numbers up to billions. On the one hand, when it comes to calculations and many of their applications – such as astronomy – their arithmetic was highly similar to ours (Ifrah, 1998). On the other hand, it had essential differences in its character. For example, the Mayan arithmetic did not include proving theorems and other aspects central to our arithmetic. This cultural variation is even more pronounced with less developed arithmetical (or proto-arithmetical) systems. Some of the numerosity systems, like the Inca *quipu* system of knots, show how versatile numerical notation can be. The variation in notations, methods, applications, sophistication, and significance of arithmetical systems is enormous, as expected in the enculturation model. One of the most striking differences concerns the number system applied. Early notations in different cultures for natural numbers share a striking similarity, showing a general tendency for tallying as the first form of keeping track of quantities. But how the tallying system of stroke notation is used and developed varies a great deal. Our decimal number system, while possibly the most common base in history, has by no means been the only one. The Mayans, Aztecs, and Celts had a vigesimal base-20 number system, whereas Sumerians and Babylonians had a base-60 system (Ifrah, 1998, xxi). Another feature central to our number system, the rule of position, has only been developed – to the best of our knowledge – on four occasions in history (Ifrah, 1998, xxiv). It is unlikely to be a coincidence that all of the four cultures (Babylonians, Chinese, Indians and Mayans) who had the rule of position – meaning that a number symbol refers to a different magnitude based on its position in the number sequence – developed sophisticated systems of arithmetic.

When considering individuals instead of whole cultures, the most important aspect seems to be simply having the cultural circumstances that provide the *opportunity* to develop arithmetical cognition. In this, arithmetic is no different from other cognitive abilities. From the *enfant sauvage* cases, it is known how difficult it is for even basic human cognitive abilities to develop without proper cultural nurturing (Candland, 1993). In the case of arithmetic, the importance of getting a suitable cultural opportunity at the right age is crucial. Regardless of their origin of birth, other than in cases of developmental disorders, children growing up in arithmetical cultures learn basic arithmetic with relative facility. This is the case also with the Pirahã children who have been raised in outside cultures (Everett, 2017).

In this way, the move from proto-arithmetic to arithmetic fits well with the theory of enculturation, having all three expected characteristics. The more we find out about the development of numerical ability, the more it points to a multitude of contributing factors, some of them genetically determined (subitizing, ANS) and some culturally specific (language, applications, cognitive tools, status). The structure of language, often thought to be the key to arithmetical cognition, would appear to be included in both.

## Whither Enculturation?

### Paradigm for Future Research

Enculturation as a general framework for explaining mathematical cognition fits well with the kind of plurality of influences described in the previous section. But the real challenge is in moving from the general idea that mathematical cognition is enculturated to satisfactory explanations of how this actually happens. This is already the case on the physiological level of neuronal recycling. Menary (2015) points out as evidence for enculturation data (e.g. Dehaene, 2009) in support of the idea that brains literally change structure and function when we learn arithmetic. Indeed, this appears to be a key point for enculturation. The whole premise of the framework is that the “mathematical brain” is not due to evolution as such, but to re-employing universal neural circuits for creating new circuits. However, enculturation is not the only framework that can account for such changes in the brain. With every piece of new information, our brain changes – that much is trivially true. The interesting question is *how* neural circuits are re-deployed.

There is thus a danger to treat enculturation as too wide a concept to be fruitful for explaining the character of arithmetical knowledge. Given the large cultural variation in how the core cognitive abilities develop into numeral systems and basic arithmetic – and further into sophisticated mathematical theories – it is easy to agree with the general culture-specific character of arithmetical conventions. However, enculturation needs to achieve more than that in order to provide a viable framework in explaining the development of arithmetic. In particular, it needs to provide a plausible account of what is specific to the development of arithmetical knowledge and how it connects to related enculturated cognitive developments, such as the development of written language.

In this pursuit, the kind of conceptual considerations I have made in this paper is crucial, starting from the distinction between proto-arithmetic and arithmetic. The enculturated character of arithmetical knowledge would be beyond our grasp if we do not have a clear idea what arithmetic *is*. As it happens, we can develop our core cognitive abilities with numerosities, as well: it is possible to become a better subitizer and estimator by training (Piazza et al., 2013). This improvement also changes the structure of the brain in an enculturated setting based on quantity-specific tasks, yet it does not make the abilities any more arithmetical. Thus, there is a clear sense in which enculturated factors influence our ability with numerosities. But in explaining arithmetical cognition, we are not interested in the general way of becoming better with quantities. Instead, the important part for us is the move from early proto-arithmetic to arithmetic and the following stages of development in arithmetical knowledge.

So far in this paper, we have focused on the initial stages in the development of arithmetic. However, that is only the beginning of a long project. The development of arithmetical thinking has been historically a slow and complicated process, and there is no reason to assume that the psychological processes carried out by individuals are straight-forward, either. After the move from proto-arithmetic to arithmetic, the next big developmental chasm is between simple counting and addition on the one hand and more developed arithmetical manipulations on the other. The former can be done before learning to write while, as Menary (2015) and Fabry (2018b) emphasize, the latter appear generally to be tied to manipulating symbols on paper (or perhaps board or screen).

From the point of view of enculturation, explaining these two stages of arithmetical development must be treated as two separate – although closely related – questions. Indeed, Menary stresses that the manipulations on paper are not scaffolding that can later be discarded. Their very essence is tied to the use of physical tools, which is a cultural aspect of its own. This embodied dimension has a crucial effect on our ability to understand mathematics. The experiments conducted by Landy and Goldstone (2007), for example, show that variation in spacing and grouping of symbols in mathematical formulas – while supposedly irrelevant to understanding the formulas – play an important role in correctly grasping operator precedence. In one experiment (Landy and Goldstone, 2009), test subjects solved algebraic equations by moving numbers to the other side of the equals sign while the background was moving. When the background moved incongruently to the correct moving of the symbols, subjects made more mistakes. Curiously, mathematically advanced students made more mistakes than beginners. This suggests how deep the importance of the sensorimotor processing can go in understanding mathematics. Even for advanced students, the physical presentation of mathematical formulas has great influence on understanding their formal content. Indeed, their processing of the formal content seems to be tied more into the particular, enculturated, procedures (see Fabry and Pantsar (2019) for more).

### Stages of Development

To make sense of what is needed in explaining the development of arithmetic, we can formulate a model of different stages in the development of arithmetic. At the first stage, from the core cognitive origins and a multitude of cultural factors, we develop a rough understanding of a discrete natural number. At the next stage, we move to counting and simple arithmetical operations with small numbers, employing new cultural inputs (such as the use of body parts, as well as numerals and number symbols). When moving to more complicated arithmetical operations, we introduce yet other cultural factors, both physical artifacts and educational innovations. This makes arithmetical operations possible for larger numbers. Ultimately and ideally, in this development, there comes the stage at which we are able to understand, construct, and communicate formal proofs of arithmetical theorems.

To apply the enculturation account in empirical studies, all these levels of the development of arithmetic need to be included, and that process involves a wide range of problems. To see why, let us consider the range of culturally dependent factors involved in learning arithmetic in Western culture. A child born into our culture shares the cognitive core systems for treating quantities with children from other cultures, but after that the surrounding culture is strongly present in every stage of the development. When she learns to count, it is made possible by her native language having a suitable numeral system. But there are also other cultural factors in play. From the educational methods to the very idea that counting is something important for young children to learn, these factors are important to acknowledge. It is not enough for a child to be, in Piaget’s (1970, 1977) terminology, in the appropriate stage of cognitive development. She also needs to receive the kind of instruction that is conducive to grasping natural number concepts (see, e.g., Ojose, 2008). Methods and contents of instruction are culturally situated inventions that determine how new knowledge is acquired, already starting from simple cases such as finger counting (Bender and Beller, 2012). This is a topic widely discussed in educational research. The sociocultural theory of Vygotsky and his followers has been used to study the early stages of mathematics education (see, e.g., Vygotsky, 1978; Case, 1987; Sfard, 2008).

Continuing to learning arithmetical operations, the cultural influence retains its central role. It is often argued that the Hindu-Arabic number symbols, for example, facilitate multiplication in comparison to, say, the Roman number symbols, thus changing the status of multiplication in the culture (Wilensky and Papert, 2010). In our culture, almost every child learns multiplication with the Hindu-Arabic number symbols. This is of course not only a question of number symbols but also of multiplication algorithms and the educational context for learning them. Saxe (2015), for example, has showed that there are important difference between the arithmetic used by child candy sellers in Brazil and that of school-educated children. Our cognitive development is determined by not only the languages and symbols systems that we use but also the other aspects of the cultural context. With the development of artifacts such as the abacus, the slide rule, or the electronic calculator, the education and practice of arithmetic are once more changed, as different cognitive tools are being employed in different cultural settings (Hutchins, 1995; Malafouris, 2013).

Assuming that the child progresses in mathematics when she grows up, she may continue to study it on a higher level. Now she will learn what kind of proofs are acceptable. She will be taught a whole new way of thinking about arithmetic. Whereas before her arithmetical efforts have involved carrying out arithmetical operations with particular numbers, now she learns how to prove theorems concerning all numbers. She will learn about formal mathematics: axiomatic systems, proof methods, etc. Equally importantly, she will also learn about mathematical *practice*: how to construct mathematical proofs and communicate them to other mathematicians (see, e.g., Avigad, 2008). And if she does not become a mathematician, she could become an engineer, an architect, or work in any of the multitude of professions in our culture that apply mathematics. All through this development, from learning to count to becoming a proficient (if not necessarily professional) mathematician, her brain will continue to recycle neural circuits to new functions, determined by the instruction and influences she receives from the surrounding culture.

Explaining this development in a comprehensive manner is a complex project when we remember that enculturation can be a different process for different stages of the development of arithmetic. Indeed, it can conceivably be in different cultures a different process also for the *same* stage. Even if our conception of, say, addition was essentially equivalent to that of the Mayans, there is no guarantee that the enculturated process leading to it was similar in the two cultures. It is this vastness of arithmetic as a human phenomenon in all its aspects – both within cultures and inter-culturally – that makes explaining the development of arithmetical cognition a wider project than traditionally thought. If we want to get an empirically informed and conceptually sound explanation of arithmetical cognition, the only possibility is to break the wider phenomenon into smaller pieces. In this paper, I have presented an enculturated account of how to do this in the move from proto-arithmetic to arithmetic.

## Author Contributions

The author confirms being the sole contributor of this work and has approved it for publication.

### Conflict of Interest Statement

The author declares that the research was conducted in the absence of any commercial or financial relationships that could be construed as a potential conflict of interest.

## Footnotes

^1^See Uller et al. (1999) for one such account. They argue that the infants’ representations are based on *object files* rather than integer representations. See section “Proto-Arithmetical Ability” for more on this.

^2^A similar distinction can be found in De Cruz et al. (2010). Based on the same type of consideration, Núñez (2017) suggests that instead of “numerical” ability, it would be better to talk about “quintical” ability when it comes to the proto-arithmetical treatment of quantities.

^3^However, there are some researchers who claim that a single estimation mechanism is enough to explain the data, see, e.g., Beran et al. (2006).

^4^Even those words are not used in a consistent manner, which has led some authors, most notably Frank et al. (2008), to interpret the Pirahã language as not having numeral words at all.

^5^Here, it is important to make the distinction between a language being recursive and it including a recursive numeral system. It is often argued (e.g., Hauser et al., 2002) that all natural languages are recursive. Everett (2005) argues that the Pirahã language is not, and the debate is still going on (see, e.g., Watumull et al., 2014). However, it is quite clear that not all natural languages have recursive numeral systems. Another important question is how recursivity has evolved. Pinker and Jackendoff (2005) have famously argued that recursivity must have preceded recursive languages, but this point has also been contested (see, e.g., Coolidge et al., 2011).

^6^It should be noted that there are examples of cultures who are proficient in navigation without developing geometry (Hutchins, 1995; Menary, 2018). The role of geometry in developing navigation thus seems quite different from that of arithmetic in developing trade.

^7^This has received support from the success of bi-lingual (Pirahã and Portuguese speaking) children in learning to count (Gelman and Butterworth, 2005).

^8^This is perhaps due to the left lateralization of neural circuitry associated with language processing in right-handed people. I thank Regina Fabry for this suggestion.

^9^This is not in contradiction with Tomasello’s (1999) theory that cultural evolution works through a “ratchet effect” which enable populations acquire and maintain new innovations. Changes in populations may cause innovations to be lost. Indeed, Tennie et al. (2009) acknowledge that even in steady populations the ratchet effect is not always persisting but is rather characterized through “relatively little loss or backward slippage” (p. 2405).
